# Supplementary material for: Quality Evaluation of Ophiopogon japonicus from Two Authentic Geographical Origins in China Based on Physicochemical and Pharmacological Properties of Their Polysaccharides
Source: Biomolecules. 2022 Oct 16;12(10):1491. doi: 10.3390/biom12101491 (PMC9599291; doi:10.3390/biom12101491)
Supplement: Supplementary file 1 [file biomolecules-12-01491-s001.zip › biomolecules-1962639-supplementary.pdf]

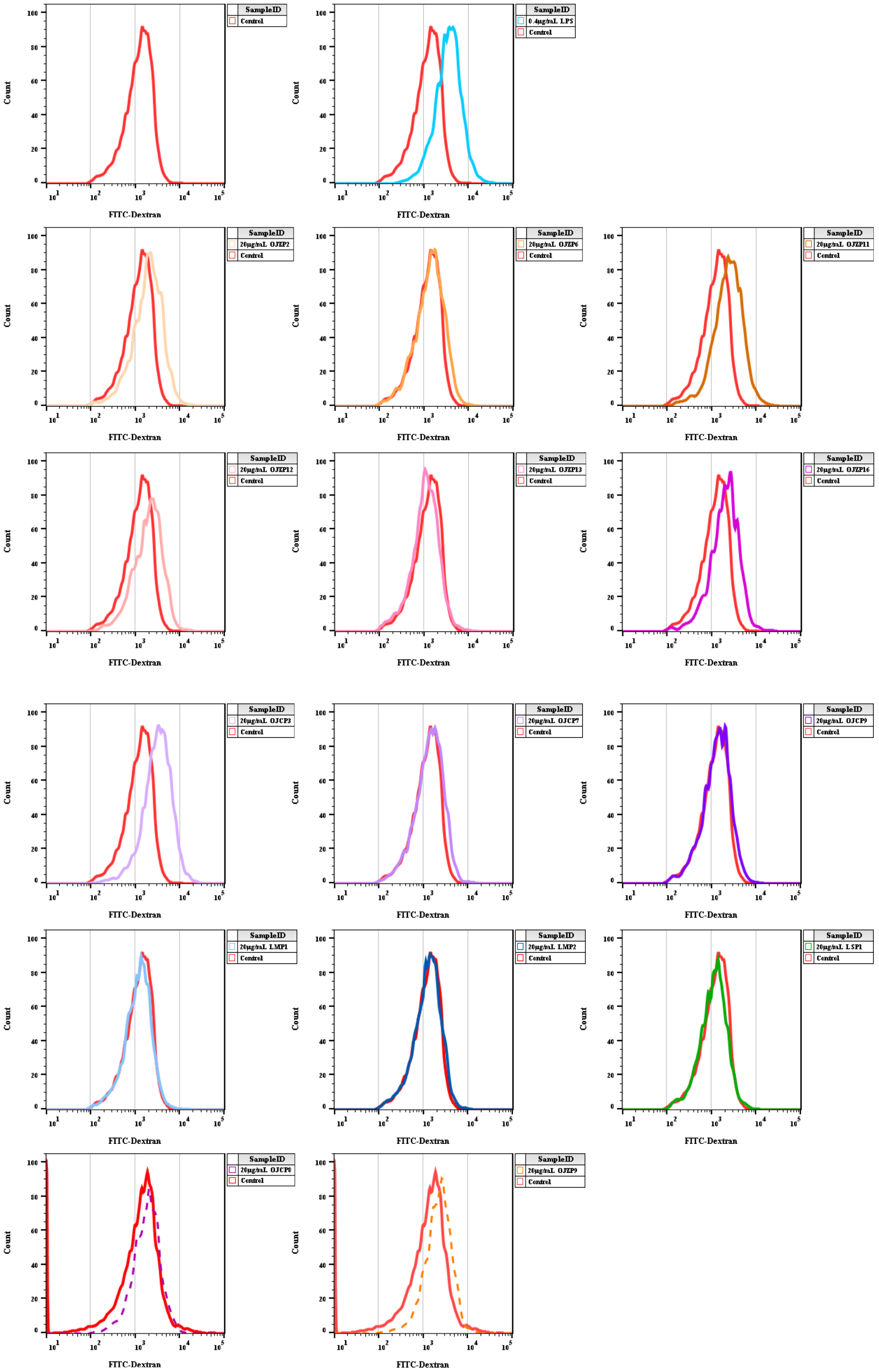

Figure S1 Typical flow cytometric profiles of phagocytosis of RAW 264.7 macrophages treated with 0.4 μg/mL of LPS, 20 μg/mL of OJZP, OJZP and LMP.

Table S1. The Pearson correlation coefficients between antioxidant/immune activity and physicochemical properties of polysaccharides in *MaiDong*.

Item		Ara	Gal	Glc	Xyl	Man	Fru	Mw-Peak1	Mw-Peak2	Mw-Peak3	DRI-Peak1	DRI-Peak2	DRI-Peak3	LS	UV-Peak2	UV-Peak3	UV-Peak4
Antioxidant activity	Pearson correlation coefficient	.832**	.863**	-.510**	.896**	.904**	-.800**	0.238	.507**	.708**	.815**	.686**	-.609**	.753**	0.151	.515**	.875**
	Sig.(2-tailed)	2.2E-08	1.8E-09	4.8E-03	5.3E-11	1.7E-11	1.9E-07	2.1E-01	5.0E-03	1.7E-05	7.2E-08	4.0E-05	4.6E-04	2.4E-06	4.3E-01	4.2E-03	5.4E-10
	Sum of squares and vector product	7.2E+01	1.1E+02	-8.3E+01	4.2E+01	9.1E+01	-2.3E+02	3.4E+06	1.3E+04	1.0E+04	6.5E-05	5.4E-04	-6.9E-04	1.2E-02	4.1E-01	1.0E+00	5.0E-01
	Covariance	2.6E+00	3.9E+00	-3.0E+00	1.5E+00	3.2E+00	-8.2E+00	1.2E+05	4.6E+02	3.7E+02	2.3E-06	1.9E-05	-2.5E-05	4.3E-04	1.5E-02	3.7E-02	1.8E-02
	N	29	29	29	29	29	29	29	29	29	29	29	29	29	29	29	29
Immune activity	Pearson correlation coefficient	.713**	.567*	-0.439	.688**	.817**	-.598*	0.229	0.207	0.442	.616*	.626*	-.657*	0.491	0.341	0.509	.659*
	Sig.(2-tailed)	4.2E-03	3.5E-02	1.2E-01	6.6E-03	3.6E-04	2.4E-02	4.3E-01	4.8E-01	1.1E-01	1.9E-02	1.7E-02	1.1E-02	7.5E-02	2.3E-01	6.3E-02	1.0E-02
	Sum of squares and vector product	1.3E+02	1.5E+02	-1.7E+02	6.6E+01	1.8E+02	-3.6E+02	6.1E+06	1.3E+04	1.7E+04	1.3E-04	1.2E-03	-1.4E-03	2.0E-02	2.1E+00	2.5E+00	6.5E-01

Covariance	1.0E+01	1.1E+01	-1.3E+01	5.1E+00	1.4E+01	-2.8E+01	4.7E+05	1.0E+03	1.3E+03	1.0E-05	9.2E-05	-1.1E-04	1.6E-03	1.6E-01	1.9E-01	5.0E-02
N	14	14	14	14	14	14	14	14	14	14	14	14	14	14	14	14

* $p < 0.05$, ** $p < 0.01$
